# Fetal Intervention in Right Outflow Tract Obstructive Disease: Selection of Candidates and Results

**DOI:** 10.1155/2012/592403

**Published:** 2012-08-15

**Authors:** E. Gómez Montes, I. Herraiz, A. Mendoza, A. Galindo

**Affiliations:** ^1^Fetal Medicine Unit-Red de Investigación en Salud Materno-Infantil y del Desarrollo (SAMID), Department of Obstetrics and Gynaecology, Hospital Universitario “12 de Octubre”, Universidad Complutense, 28041 Madrid, Spain; ^2^Pediatric Heart Institute-SAMID, Department of Pediatrics, Hospital Universitario “12 de Octubre”, Universidad Complutense, 28041 Madrid, Spain

## Abstract

*Objectives*. To describe the process of selection of candidates for fetal cardiac intervention (FCI) in fetuses diagnosed with pulmonary atresia-critical stenosis with intact ventricular septum (PA/CS-IVS) and report our own experience with FCI for such disease. *Methods*. We searched our database for cases of PA/CS-IVS prenatally diagnosed in 2003–2012. Data of 38 fetuses were retrieved and analyzed. FCI were offered to 6 patients (2 refused). In the remaining it was not offered due to the presence of either favourable prognostic echocardiographic markers (*n* = 20) or poor prognostic indicators (*n* = 12). *Results*. The outcome of fetuses with PA/CS-IVS was accurately predicted with multiparametric scoring systems. Pulmonary valvuloplasty was technically successful in all 4 fetuses. The growth of the fetal right heart and hemodynamic parameters showed a Gaussian-like behaviour with an improvement in the first weeks and slow worsening as pregnancy advanced, probably indicating a restenosis. *Conclusions*. The most likely type of circulation after birth may be predicted in the second trimester of pregnancy by means of combining cardiac dimensions and functional parameters. Fetal pulmonary valvuloplasty in midgestation is technically feasible and in well-selected cases may improve right heart growth, fetal hemodynamics, and postnatal outcome.

## 1. Introduction

Pulmonary atresia-critical stenosis with intact ventricular septum (PA/CS-IVS) is a complex and heterogeneous disease which, depending on the morphology and function of the tricuspid valve (TV), may be associated with underdevelopment of the right ventricle (RV) [1, 2] or with cardiac failure secondary to massive tricuspid regurgitation (TR) in the setting of thin-walled and dilated RV [3]. Between these two extremes of severity, the RV may show near-normal dimensions, morphology, and function, and the patient may have an adequate hemodynamic situation despite severe TR fairly due to unrestrictive interatrial shunting. Different postnatal treatment strategies are available accordingly in this wide anatomical and clinical spectrum, the ultimate aim being the achievement of biventricular (BV) circulation in an otherwise healthy patient. However, this may not be possible when the RV is severely small or when the neonate is severely sick [4]. Postnatal management of these patients may be further complicated by the presence of anomalies of the coronary arteries which are commonly seen in this disease. As a consequence, the one-year survival rate in live born infants with PA/CS-IVS is close to 70–75% [5–8] and the rate of BV outcome in survivors is around 30–50% [9–11].

Current development of fetal echocardiography and interventional cardiac technology makes it possible to offer an intrauterine approach to some cardiac diseases [12]. For PA/CS-IVS, fetuses who are likely to be suitable only for univentricular surgical palliation after birth might be selected for fetal cardiac intervention (FCI), trying to rescue right ventricular function and improve the options for BV circulation. Similarly, in those with severe hemodynamic compromise, the evolution to fetal hydrops and fetal-neonatal death may be halted by means of FCI. Indeed, some successful cases of fetal pulmonary valvuloplasty have been reported [13, 14]. However, this procedure also carries a risk of fetal morbidity and mortality, making it necessary to properly balance the risks and likely benefits of FCI in every single case. Therefore, differentiation of fetuses situated at the worst end of the spectrum of PA/CS-IVS from more favourable cases, which can be managed expectantly, is of paramount importance.

The objective of this paper is to describe firstly the process of selection of candidates for FCI in fetuses diagnosed with PA/CS-IVS and secondly report our own experience with FCI for such disease.

## 2. Methods

Our database was queried for cases of PA/CS-IVS prenatally diagnosed in the period October 2003–January 2012 at our tertiary care referral centre. PA was diagnosed by the absence of flow across the pulmonary valve and CS by the presence of thickened and domed pulmonary valve cusps with a pinhole jet of flow, and in both with Doppler evidence of ductal-dependent pulmonary circulation [2]. Fetuses with functional PA (e.g., in the setting of Ebstein's anomaly with severe TR) were excluded.

Fetal ultrasound examinations included a detailed extracardiac structural survey and a complete echocardiography, performed following guidelines [15, 16]. All scans were performed by experienced obstetricians using high-quality machines, and all cases were analyzed together with a pediatric cardiologist. The methodology for the morphological and functional assessment of all cases has been described elsewhere [17]. The *z*-scores for most cardiac dimensions were obtained with the software available [18]. The presence of ventriculocoronary connections (VCCs) was routinely assessed in every case.

### 2.1. Selection of Patients with PA/CS-IVS for Fetal Cardiac Intervention

All fetuses diagnosed as having PA/CS-IVS routinely undergo an echocardiographic assessment aimed to predict their more likely outcome (BV versus non-BV circulation) and also to evaluate the risk of developing right heart failure.

#### 2.1.1. Predicting Univentricular Outcome

Different criteria for predicting non-BV outcome have been described over time (Table 1). In our clinical setting, the prediction was based always on multiparametric criteria [19], and recently we have described our own scoring system, which is the one that we are currently using [17]. FCI was offered when non-BV outcome was predicted and favourable anatomy was present, which implicates that the outlet portion of the right ventricle may have thin membranous atresia with an open outflow tract below the valve and with reasonable sized pulmonary arteries.

#### 2.1.2. Predicting Right Heart Failure

In these cases the decision on whether performing FCI relied upon the assessment of the parameters which are commonly evaluated in fetuses with PA/CS-IVS and marked TR, including cardiac size, severity of TR, venous Doppler, and peripheral edema, as suggested by Huhta [20]. We consider FCI whenever progressive and severe worsening of these parameters was observed in fetuses far from viability in order to prevent the potential development of hydrops and fetal death.

### 2.2. Technique of Fetal Cardiac Intervention

**Figure 1:**
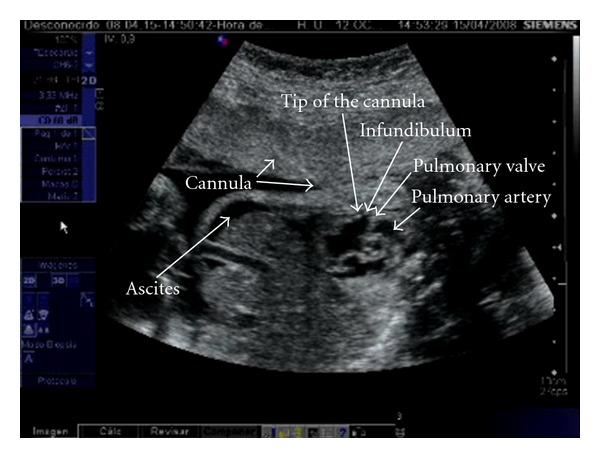
Cannula course during fetal cardiac intervention for pulmonary valvuloplasty, entering the right ventricle at the infundibulum, in the longitudinal view, with the cannula course parallel to the RV outflow tract.

**Figure 2:**
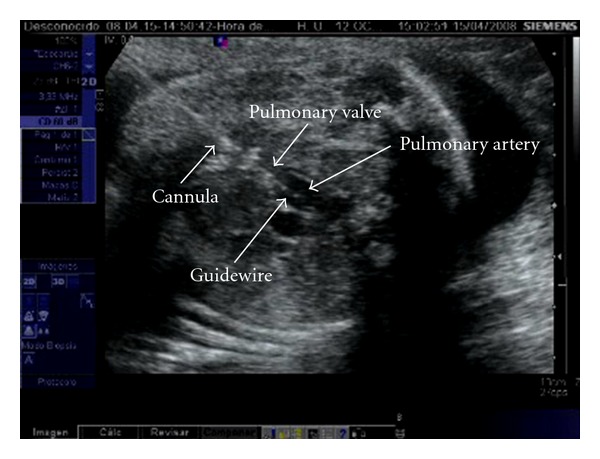
The guidewire has crossed the pulmonary valve, and it is inside the main pulmonary artery.

**Figure 3:**
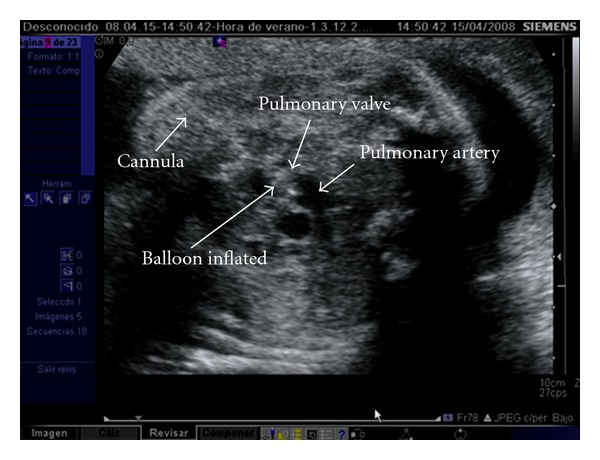
The coronary angioplasty balloon is inflated across the fetal pulmonary valve.

First, both antibiotic prophylaxis with cefazolin and premature delivery prophylaxis with indomethacin are administered. Only the first fetal pulmonary valvuloplasty was performed under general anesthesia, in order to provide more uterine relaxation, thus allowing manipulation of the fetus into a favourable position. The rest has been performed under maternal local anesthesia together with sedation and administration of opioid analgesics. An injection of atropine (0.1 mg), vecuronium (0.2 mg/kg), and fentanyl (20 *μ*g/kg) is administered intramuscularly to the fetus for paralysis and anesthesia before passage of the cannula. Prior to FCI, the cannula, guidewires, and balloon shafts are premeasured and marked so that positioning within the heart is known from external measurements rather than the ultrasound imaging alone. Under continuous ultrasound guidance, we advanced a 15 cm long 18-gauge cannula and stylet needle through the maternal abdomen into the uterus and from there through the fetal chest wall into the cardiac region of interest. Specifically, the RV is entered at the infundibulum, in the longitudinal view, with the cannula course parallel to the RV outflow tract, making easier thereby crossing the valve with the device (Figures 1 and 2). Following the appropriate positioning of the needle in the infundibulum, a floppy-tipped guidewire of 0.014 inch is passed through the stenotic valve, and in cases of PA the valve is perforated with the stylet of the needle. Once the guidewire is in the pulmonary artery, a coronary angioplasty balloon is inflated across the valve, with balloon/valve ratio diameter of 1.2 (Figure 3). Three to five inflations are done to assure that the valve has been appropriately enlarged. In case of sustained fetal bradycardia, immediate intracardiac injection of atropine is used, and hemopericardium is aspirated if present. Technical success is defined as successful passage and inflation of a dilating balloon across the pulmonary valve, with subsequent improvement in antegrade flow on color Doppler imaging with or without pulmonary regurgitation. After the final balloon deflation, the entire device is removed.

### 2.3. Postprocedural Follow-Up

We monitor the growth and function of the fetal heart in all subjects. Serial follow-up evaluations every 3-4 weeks are performed until delivery. Follow-up echocardiograms include the assessment of growth of the right heart structures and central and peripheral pulsed and color Doppler flow patterns. Depending on which is the dominant source of flow of the pulmonary artery, the filling is classified as antegrade (the RV), reversed (the arterial duct), or mixed (both equally).

### 2.4. Postnatal Management

Our standard practice is to perform an echocardiogram and cardiac catheterization in the first days of life. Patients with severely small RV (uni- or bipartite, TV *z*-score < −5) or with RV-dependent coronary circulation, defined following published criteria [21], are included in a single ventricle palliation pathway, and they undergo firstly a systemic-to-pulmonary shunt in the neonatal period, followed by a bidirectional Glenn anastomosis which is performed around 6 months of age and finally a Fontan operation at 3 years of life. The remaining neonates are included in the BV management pathway: they undergo a pulmonary valvotomy, which is attempted firstly with transcatheter balloon and if this fail is performed surgically, that may be associated with systemic-to-pulmonary artery shunt depending on the size of the RV and TV. Further reevaluation is performed at 6 months of age to assess if the RV is able to support the pulmonary circulation, which implies closing the systemic-to-pulmonary artery shunt or not. In this last situation a bidirectional Glenn operation is performed, and these patients undergo a new cardiac evaluation between 1 and 3 years of age in order to determine whether a 1.5 ventricle repair or Fontan operation is appropriate.

A BV repair was defined as a RV finally supporting the pulmonary circulation, a systemic arterial saturation >90%, and either no interatrial communication or any remaining left-to-right shunt. Otherwise, patients were considered to have a non-BV outcome.

Postnatal outcome was ascertained through our own hospital medical records. Postnatal follow-up for at least 12 months was available for all the surviving patients.

## 3. Results

**Figure 4:**
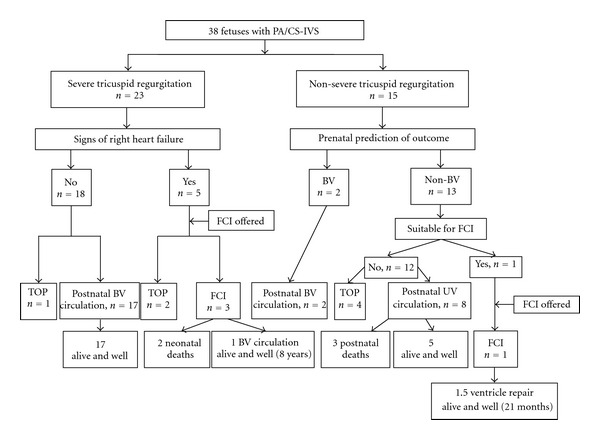
Flow chart showing the distribution of fetuses with pulmonary atresia/critical stenosis with intact ventricular septum (PA/CS-IVS) according to the process of selection for fetal cardiac intervention (FCI). BV, biventricular; UV, univentricular; TOP, termination of pregnancy.

During the study period the diagnosis of PA/CS-IVS was established in 38 fetuses—18 with PA and 20 with CS. The mean gestational age at diagnosis was 23.1 ± 3.5 weeks. Most fetuses showed severe TR (23/38, 61%), and 5 of them also had a cardiovascular profile score <7. FCI was offered in all 5, and it was accepted in 3. The rest of the fetuses presented with non-severe TR, and in most of them a non-BV outcome was predicted. Only one fetus was considered suitable to FCI at the time of diagnosis and it was accepted. FCI was not offered in 32 fetuses: in 7 cases the parents opted to terminate the pregnancy, and among those surviving the perinatal period their outcome was accurately predicted prenatally. The prenatal diagnosis was confirmed after birth by echocardiographic and angiographic examinations in all patients.  Figure 4 summarizes the distribution and outcome of the whole group.

### 3.1. Fetal Pulmonary Valvuloplasty

Four fetuses underwent pulmonary valvuloplasty, performed at a mean gestational age of 25 weeks. All cases were performed with percutaneous access and were technically successful. The first three cases were diagnosed as having CS-IVS, with a pinhole jet of flow (1 mm) across the PV, while the fourth was diagnosed of PA-IVS. Baseline and follow-up echocardiographic data of the 4 cases as well as their outcome are summarized in Table 2.

**Case 1**

Associated with the data shown in Table 2, this fetus showed progressive cardiomegaly (CTI from 62% at 23 weeks to 71% at 25 weeks) and rapid worsening of ductus venosus Doppler waveform (from small but antegrade flow at 23 weeks to clearly reversed in all cardiac cycles at 25 weeks). The procedure resulted in antegrade flow across the PV with a velocity of 1.1 m/s. Follow-up scans showed normal ductus venosus waveform and antegrade flow across the PV. TR velocity fell to 1.8 m/s. Right heart dimensions were consistently normal and showed a slight improvement in comparison with pre-FCI. At 34 weeks the pulmonary flow was again almost exclusively ductus-dependent with reversed diastolic flow in the ductus venosus, consistent with a TR velocity of 2.8 m/s, a probable indicator of restenosis. After fetal lung maturity testing, a cesarean section was performed at 34 + 3 weeks delivering a male newborn weighing 2340 g. Neonatal echocardiographic examination confirmed prenatal findings showing a tripartite RV with appropriate TV and PV, with a tiny hole across the pulmonary leaflets. A cardiac catheterization was scheduled at 72 h of age during which a successful pulmonary valvuloplasty was performed. Ten months later the infant underwent an open valvotomy and now at the age of 7 the patient is asymptomatic, medication-free with RV size and function all within normal limits, despite moderate pulmonary regurgitation.

**Case 2**

Following the initial diagnosis of a dysplastic pulmonary valve at 22 weeks, this fetus developed ascites likely due to restrictive right-to-left interatrial shunting of 1.5 mm, accompanied by cardiomegaly (CTI 58%), mild pericardial effusion, and high velocity TR (3.1 m/s). A forward jet of flow of 2.6 mm was observed immediately after valvuloplasty, with a peak systolic velocity of 0.9 m/s and a holodiastolic pulmonary regurgitation. Color Doppler showed a dominant antegrade filling of the central pulmonary arteries. Initial follow-up scans also showed a slight reduction in the TR velocity (1.5 m/s), an improvement in venous Doppler with antegrade end-diastolic flow in the ductus venosus and a reduction in the amount of ascites. These features were stable throughout the pregnancy, the antegrade jet of flow across the PV reached a maximum diameter of 4.5 mm, the right heart dimensions remained within normal limits, the RV showed normal tripartite appearance with myocardial hypertrophy affecting the trabecular component, but in the last scans before birth TR velocity rose again to 2.8 m/s. Spontaneous preterm delivery of a male weighing 2600 g took place at 35 weeks. Postnatal echocardiography confirmed the prenatal diagnosis. Diagnostic catheterization performed 48 h after birth showed VCCs in the RV and interruption of the circumflex artery. During this procedure the patient became hemodynamically unstable and had an episode of cardiac arrest. Diffuse ischemic lesions were seen in the central nervous system three days later, and the therapeutic support was subsequently withdrawn.

**Case 3**

Following the initial diagnosis at 21 weeks of CS-IVS with a right coronary to RV fistula, the fetus remained in a stable condition with tripartite normal-sized RV, a small PV, high-velocity holosystolic TR (2 m/s), and absent-reversed diastolic flow in the ductus venosus. Therefore, the patient was not considered initially to be a candidate for FCI. However, a new scan performed at 26 weeks showed the presence of pericardial effusion and ascites. A forward jet of flow of 2.3 mm was observed immediately following valvuloplasty, with a peak systolic velocity of 1.2 m/s, mild protodiastolic pulmonary regurgitation, and mixed filling (antegrade and duct dependent) of the pulmonary artery. This situation remained stable in the initial follow-up scans, but it was not accompanied by changes in venous Doppler, in the severity of TR, in the flow waveform in the fistula, or in the amount of ascites. Right heart dimensions maintained within normal limits and the RV kept its normal tripartite appearance, with myocardial hypertrophy affecting the trabecular component. This leads us to hypothesize that pulmonary valvuloplasty could at least halt the deterioration of the fetus, preventing the progression of right heart failure. With a similar antegrade jet of flow across the pulmonary valve of 2.6 mm and peak systolic velocity of 1.17 m/s, a relative worsening was observed at 33 weeks including small growth of the RV, increasing ascites and reappearance of a small amount of pericardial fluid. It was considered that early delivery would improve the chances of biventricular repair, and after induction of fetal lung maturity, labor was induced at 34 + 2 weeks. Unfortunately, delivery was complicated by intrapartum placental abruption, and despite urgent caesarean section, a severely hypoxemic baby weighing 2000 g was born and died at 24 hours secondary to multisystem organ failure.

**Case 4**

Following the initial diagnosis at 21 weeks of PA-IVS, this patient was referred to our unit at 24 weeks. Fetal echocardiography showed a tripartite but small RV, which did not reach the apex, with myocardial hypertrophy affecting the trabecular component and biphasic filling followed by holosystolic TR with peak velocity of 1.5 m/s. In such a scenario, a BV outcome was considered highly unlikely and pulmonary valvuloplasty was attempted 3 days later. A forward jet of flow of 2.3 mm was observed immediately after valvuloplasty, with a peak systolic velocity of 1.4 m/s, mild protodiastolic pulmonary regurgitation, and mixed filling (antegrade and duct dependent) of the pulmonary artery. These features remained stable in follow-up scans together with biphasic RV inflow and increased TR, which was of high velocity (2.4 m/s), likely reflecting an improvement in RV filling. However, no significant changes were seen in venous Doppler throughout the pregnancy. The anatomy was favourable with a good-sized RV and TV, increasing thereby the chances for a BV outcome. However, between 32 and 37 weeks the *z*-score of the TV fell from −1.8 to −2.7 and the RV/LV ratio decreased from 0.60 to 0.52. This child was delivered by elective caesarian section for a previous caesarian section at 38 weeks, weighing 2630 g. Postnatal echocardiography showed a small tripartite RV, and after a failed pulmonary valvuloplasty, an open valvotomy with placement of a modified right Blalock-Taussig shunt was performed at the age of 2 weeks. This girl has undergone a one and a half ventricle repair and is healthy at 21 months.

## 4. Discussion

More than 20 years have elapsed since the first description of a FCI [23], and the role of this type of procedures in fetal cardiology is still a matter of controversy. Certainly, most CHDs can now be repaired successfully in the postnatal period with excellent results in terms both of mortality and long-term outcome. Therefore, for these defects there would be no need for fetal intervention. However, it is clear that the evolving nature of some cardiac diseases like the severe semilunar valvar stenosis often exposes the affected patient to a significant morbidity and mortality [9–11, 24, 25], and therefore there seem to be good clinical reasons to further attempt prenatal cardiac interventions aiming to modify their natural history. Moreover, there is evidence that the restoration of normal flow promotes growth and the reduction in ventricular pressure enables more normal development and function [26–28]. Once the cardiac defects with the potential to be intervened in utero are established and the rationale for intervention seems to be strong enough, two major points should be addressed before attempting FCI: firstly, the accurate selection of the fetuses with PA/CS-IVS that might certainly benefit from FCI and secondly, the feasibility, safety, and results of FCI.

Regarding the first point, several predictive models have been described in the last years. In accordance with the policy usually followed postnatally, where the decision regarding the management pathway usually relies upon several cardiovascular parameters which address both the size of the right inflow and outflow tracts and morphological features as well [29–31], we have observed that the performance of multiparameter scoring systems [12, 17, 19] is better than single-parameter models [22]. Certainly, in our experience the most likely type of circulation after birth can be accurately predicted in the second trimester of pregnancy by means of a combination of three size-based parameters (TV/MV ratio, RV/LV length ratio, pulmonary valve/aortic valve ratio) and one functional marker addressing the RV preload (tricuspid inflow duration/cardiac cycle length). If 3 of these 4 markers are present in a fetus diagnosed with PA/CS-IVS ≤ 28 weeks of gestation, this predicts a non-BV outcome with sensitivity of 100% and specificity of 92%, and these two are 100% if the 4 criteria are fulfilled. These markers are objective measurements less prone to errors than the subjective assessment of the morphology of cardiac structures and showed to have the best balance between sensitivity and specificity for predicting non-BV circulation after birth, avoiding false negative results which would imply not selecting potential candidates to fetal therapy. Therefore, despite the potential for progression of this cardiac disease [32], the initial surgical pathway can be established in the first diagnostic scan, which is usually made in the mid-second trimester. This is of paramount importance for (i) early selection of potential candidates for FCI, when the chances for a successfully right-sided cardiac salvage are maximal and (ii) for early parental counseling providing them with valuable information which is relevant in the decision-making process.

In the setting of severe TR, it is of paramount importance the analysis of surrogate markers of right heart failure. In this sense either morphological parameters such as serous effusions, skin edema, or severe cardiomegaly or hemodynamical markers such as the severity of TR and ductus venosus flow may provide useful information regarding the degree of raised systemic venous pressure [33, 34]. Specifically, if the sequential interrogation of the ductus venosus flow in fetuses with PA-CS/IVS, which can easily be made in most instances, shows progressive worsening of the reversed a-wave and this pattern is observed in combination with other signs of right heart failure, it should be interpreted as a definitive sign of cardiac compromise, probably leading to an adverse outcome [12–14, 35].

FCI for patients affected with PA/CS-IVS is a challenging procedure given the special anatomy of the right outflow tract and the difficult access to the pulmonary valve. However, it has been shown that pulmonary valvuloplasty in fetal life is technically feasible even in mid-gestation, although it should be acknowledged that the experience with this procedure is still limited to a few number of cases [13, 14, 35]. Regarding the safety of fetal pulmonary valvuloplasty, our results show that it may be safely performed both for the mother and the fetus provided that they are made by an experienced team in fetal invasive procedures. This is of paramount importance given that although this type of procedure carries a risk of fetal mortality and morbidity, the accumulated experience seems to demonstrate that the fetus tolerates better FCI for right outflow tract diseases than for the aortic valve, probably attributable to the fact that there are no coronary arteries to be occluded during balloon inflation. Our 4 fetuses underwent technically successful pulmonary valvuloplasty, and this was followed by a slight but recognizable improvement in the right heart growth and hemodynamics during the early weeks after the procedure. However, we and others have observed that the duration of this effect is variable but almost always is transient, giving a Gaussian-like evolution for most of the parameters. Unfortunately, this prenatal improvement not always is followed by the desirable outcome: in fact, two of our patients died shortly after birth although in only one this could be explained by the cardiac disease itself. Nevertheless, this procedure may change the adverse natural history of PA/CS-IVS and allows for fetal maturity to be reached in better conditions. Therefore, we firmly believe that there is a place for fetal pulmonary valvuloplasty provided that an accurate selection of suitable cases is made and the procedure is performed under low-risk conditions, that is, in experienced centers.

In conclusion, although we acknowledge that there is still limited experience with fetal pulmonary valvuloplasty and that randomized studies have not been performed (and probably never will), it seems that in selected cases this intervention may improve fetal hemodynamics, right heart growth, and postnatal outcome. Future experience will give us a better knowledge about the prenatal and postnatal outcome of FCI in right cardiac defects.

## Figures and Tables

**Table 1:** Criteria used for prenatal echocardiographic prediction of postnatal outcome for pulmonary atresia/critical pulmonary stenosis with intact ventricular septum (PA/CS-IVS).

Reference	Prenatal predictors of non-BV outcome	Test accuracy
Salvin et al. (2006) [22]	(i) TV *z*-score ≤ -3	Sn 92%, Sp 100%
Roman et al. (2007) [19]	(i) TV/MV ratio < 0.7	3/4 criteria: Sn 100%, Sp 75%
	(ii) RV/LV length ratio < 0.6	
	(iii) Tricuspid inflow duration/cardiac cycle length ≤ 31.5%	
	(iv) Presence of ventriculocoronary connections	
Gardiner et al. (2008) [12]	(i) PV *z*-score < −1 or TV *z*-score < −3.4 before 23 weeks	(i) 2/2 criteria < 23 weeks: Sn 100%, Sp 80%
	(ii) Median TV *z*-score < −3.95, before 26 weeks	(ii) 1 criteria < 26 weeks^∗^: Sn 92%, Sp 100%
	(iii) Median PV *z*-score < −2.8 and medium TV/MV ratio < 0.7 at 26–31 weeks	(iii) 2/2 criteria at 26–31 weeks: Sn 100%, Sp 100%
	(iv) Median TV *z*-score < −3.9 and medium TV/MV ratio < 0.59 after 31 weeks	(iv) 1 criteria after 31 weeks: Sn 100%, Sp 100%
Gómez-Montes et al. (2011) [17]	(i) TV/MV ratio ≤ 0.83	
	(ii) RV/LV length ratio ≤ 0.64	4/4 criteria: Sn 100%, Sp 100%
	(iii) PV/AV ratio ≤ 0.75	3/4 criteria: Sn 100%, Sp 92%
	(iv) Tricuspid inflow duration/cardiac cycle length ≤ 36.5%	

TV: tricuspid valve; MV: mitral valve; RV: right ventricle; LV: left ventricle; PV: pulmonary valve; AV: aortic valve; BV: biventricular; Sn: sensitivity; Sp: specificity.

^∗^In combination with hemodynamical markers of right atrial pressure (severity of tricuspid regurgitation, waveform characteristics of the ductus venosus, and restriction of the interatrial septum) and the presence of coronary artery fistulae.

**Table 2:** Echocardiographic data before and after prenatal pulmonary valvuloplasty for PA/CS-IVS.

Case	CHD	GA at diagnosis/ at FCI, weeks				Before FCI												After FCI									Outcome
														At 2-4 weeks								At 6–8 weeks					
			*z*-score			RV/LV ratio	Flow				*z*-score			RV/LV ratio	Flow				*z*-score			RV/LV ratio	Flow				
			TV	PV	PA		AD	DV	TR	TR, m/s	TV	PV	PA		AD	DV	TR	TR, m/s	TV	PA	PA		AD	DV	TR	TR, m/s	
1	CS-IVS	23/25	−0.6	−2.4	2.3	0.67	Rv	Rv	Sv	2.4	1.0	−1.4	1.6	0.64	At	At	Sv	2	1.5	1.5	1.0	0.71	Rv	Rv	Not Sv	2.8	Alive and well
																											Biventricular circulation
2	CS-IVS	22/25	−1.0	0.2	1.7	0.44	Rv	Rv	Sv	3.1	−1.6	−0.2	0.7	0.56	At	At	Not Sv	1.5	−1.7	0.9	2.5	0.56	At	Ab/Rv	Not Sv	2.8	Postnatal death
3	CS-IVS	21/26	−0.3	−3.4	−0.9	0.53	Rv	Rv	Sv	2	0.1	−1.9	−0.7	0.54	Mx	Rv	Sv	2	−3.4	−3.6	−1.2	0.57	Mx	Rv	Not Sv	2	Postnatal death
4	PA-IVS	21/24	−4.4	−1.9	−0.9	0.51	Rv	Rv	Sv	1.5	−1.6	0.0	−1.5	0.63	Mx	Rv	Sv	2.4	−2.7	−1.4	−0.9	0.52	Mx	Rv	Sv	1.4	Alive and well One
																											and a half ventricle repair

CHD: congenital heart defect; GA: gestational age; FCI: fetal cardiac intervention; TV: tricuspid valve; PV: pulmonary valve; PA: pulmonary artery; RV/LV ratio: ratio between the length of both ventricles, measured in early systole in the four-chamber view; AD: arterial duct; DV: ductus venosus; TR: tricuspid regurgitation (it was considered severe—Sv—if it was of holosystolic, reaching the wall of the right atrium. Otherwise, it was considered not severe—Not Sv); Rv: reversed; At: antegrade; Mx: mixed; Ab: absent.
